# Do Peer Cliques and Gender Differences Shape Adolescent Depression Under Bullying? Exploring the Mediating Power of Cognitive Biases

**DOI:** 10.3390/bs16010068

**Published:** 2026-01-04

**Authors:** Xingyuan Wang, Caina Li, Tianyang Wang

**Affiliations:** 1 School of Elementary Education, Changji University, Xinjiang Research Center of Improving Basic Education Quality, Changji 830011, China; 2 School of Psychology, Shaanxi Normal University, Shaanxi Provincial Key Research Center of Child Mental and Behavioral Health, Xi’an 710062, China; chinali@snnu.edu.cn (C.L.); wty026027@163.com (T.W.)

**Keywords:** clique norms, victimization, cognitive biases, depression, depressive symptoms, adolescents, gender differences

## Abstract

The Healthy Context Paradox suggests that victims of bullying struggle more with psychological adjustment in environments with low victimization norms. This study, guided by Beck’s Model of Depression, explores this phenomenon through a cognitive lens. Using data from 2091 Chinese junior high students (54.3% boys, mean age 13.26), we identified cliques via the Social Cognitive Map and examined the mediating role of cognitive biases and the moderating role of clique-level victimization norms in the link between peer victimization and depressive symptoms. Results showed that cognitive biases partially mediated the link between peer victimization and depressive symptoms. While clique victimization norms moderated the association between peer victimization and cognitive biases, they had no significant relation to depressive symptoms. In low victimization norm cliques, peer victimization showed a stronger association with cognitive biases, especially in all-girl cliques, whereas this association was observed in all-boy cliques irrespective of norms. The moderating effects of clique victimization norms on the association between peer victimization and depressive symptoms were non-significant in all-boy and mixed-gender cliques. These findings suggest that integrating the Healthy Context Paradox with Beck’s Model can inform depressive symptom prevention strategies, particularly in bullying-prone environments.

## 1. Introduction

The phrase “Once a victim, always a victim” highlights the pervasive nature of peer victimization, a common phenomenon persisting from adolescence into adulthood that is consistently linked to multiple negative outcomes, including but not limited to depression (Hawker & Boulton, 2000). Previous studies have demonstrated that peer victimization and depression are closely associated with socio-cognitive factors such as self-esteem and rumination (van Geel et al., 2018), and individuals with depression exhibit more severe biased cognitive processes (Nieto et al., 2020). Nevertheless, the relationships among the three variables have not been fully explored in prior research.

Bullying often occurs in contexts where clique norms significantly influence behavior, values, and expectations (Zhao & Li, 2022). These norms directly impact members’ acceptance of bullying and the psychological adjustment of victims. Recent studies suggest that depression levels may vary under different clique victimization norms (Pan et al., 2021). To better understand the relationship between peer victimization and depression, this study innovatively examines the mediating effect of cognitive biases, with clique victimization norms as a moderator.

### 1.1. Depression and Victimization

Depression is a complex mental disorder that, according to the World Health Organization’s 2023 Global Burden of Disease Report, affects 3.8% of the global population, corresponding to approximately 280 million people, and exhibits a higher prevalence among women (6%) than men (4%). It significantly impacts adolescent mental health, often leading to negative coping styles (Giles & Shaw, 1987). The incidence of depression rises from childhood to adolescence, a period which coincides with peak bullying victimization (Kessler et al., 2001). Strong links between depression and peer victimization are well-documented, especially in adolescents aged 11–15 years (Klomek et al., 2008; Sweeting et al., 2006).

While most research focuses on external social consequences of victimization, few studies explore the internal mechanisms connecting peer victimization to depression. Current research also prioritizes social contextual changes over the cognitive factors that contribute to increased depressive symptoms. This study aims to investigate the association between cognitive biases in bullying victims and their depressive symptoms, offering insights into the internal processes involved.

### 1.2. The Effects of Cognitive Biases: Beck’s Model

Cognitive biases, which involve distorted perception of reality shaped by problematic social interactions or circumstances, are strongly linked to childhood depression (Li et al., 2013; Nieto et al., 2020). Beck’s cognitive model of depression identifies dysfunctional thinking or cognitive biases as a key factor in emotional disorders like depression (Beck, 2008). As mechanisms of the cognitive system, these biases affect memory, attention, and interpretation. This leads to distorted views of self, others, and the world. These biased processes are considered a core contributor to depression. A meta-analysis by Nieto et al. (2020) further confirms that individuals with depression exhibit stronger cognitive biases. Empirical studies also support the link (Everaert et al., 2012), offering a perspective from Beck’s model to explain how cognitive biases correlate with depression, particularly following victimization.

Previous research has shown that victimization experiences can impact children’s cognitive processing, specifically by activating vulnerable schemas (Rosen et al., 2007). These schemas, which guide social interactions (Baldwin, 1992), make children more responsive to social threats. Once activated, these schemas influence memory, attention, and interpretation, which can lead to cognitive biases that contribute to depression. Specifically, victimized adolescents exhibit negative encoding and interpretation of social cues along with negative thinking patterns (Cole et al., 2014; Kellij et al., 2022). Longitudinal studies further confirm that early victimization is associated with negative cognitive biases in adolescents (Cole et al., 2016; McDougall & Vaillancourt, 2015; Wolke & Lereya, 2015). Negative schemas originating from early victimization tend to persist over time (Calvete et al., 2018), as accumulated victimization repeatedly activates these schemas, routinizing maladaptive information processing patterns. Such negatively biased cognitive schemas function as automatic information processors (Beck, 2008). Early victimization shapes information processing, modifies attention or memory functions, and thereby fosters negative cognitive biases toward current experiences (Bomyea et al., 2017). Thus, cognitive biases serve as a critical mechanism bridging peer victimization and depressive symptoms.

### 1.3. The Moderating Role of Clique Victimization Norms and Unresolved Theoretical Questions

Victimization, a pervasive issue, often occurs within close-knit groups, or cliques, in a larger social context. This can lead to the establishment of clique victimization norms. These norms are defined as the average level of victimization within a clique (Zhao & Li, 2022) and represent the social expectations regarding how bullying and victims are perceived by group members and outsiders. Moreover, in cliques with lower victimization (i.e., “healthier” contexts), instances of peer victimization are more noticeable. In such settings, victims report lower self-esteem and more depressive symptoms (Huitsing et al., 2019), potentially because the experience feels more humiliating and isolating.

This phenomenon is articulated through the Healthy Context Paradox, which suggests that victims in low victimization settings may encounter heightened social adjustment difficulties. Within such contexts, victims are more likely to develop negative self perceptions and face reduced peer acceptance (Zhao & Li, 2022). Consistent with this, studies have found that victimized individuals tend to exhibit more depressive symptoms in cliques where victimization is infrequent (Pan et al., 2021). This process can be understood through Beck’s cognitive model of depression, which identifies negative self schemata as a core form of cognitive bias that links adverse experiences to depressive symptoms. In low victimization norm contexts, the discrepancy between individual victimization experiences and prevailing group norms may intensify such cognitive biases, thereby clarifying the psychological mechanism behind the Healthy Context Paradox. In contrast to the “Healthy Context Paradox”, the peer contagion hypothesis posits that victims in high-victimization small cliques exhibit greater deviant emotions and behaviors compared to those in low-victimization small cliques (Dishion et al., 1996; Dishion & Tipsord, 2011; Dodge et al., 2006). This theory suggests that high-aggression norms prompt clique members to engage in “deviant behavior training” (Dishion & Tipsord, 2011) or social learning (Bandura, 1973). Consequently, individuals’ emotions and behaviors become more susceptible to peer influence in highly victimizing environments. This process may intensify their depressive symptoms, potentially through reinforced negative cognitive patterns. These two competing frameworks thus raise a critical unresolved question: Do clique victimization norms alleviate or exacerbate the association between peer victimization, cognitive biases, and depressive symptoms? To address this theoretical ambiguity, the current study aims to examine the role of cognitive biases as a mediator in the link between peer victimization and depressive symptoms, while also investigating how clique-level victimization norms moderate this chain of relationships.

Prior multilevel research has established that clique norms can act as predictors or moderators of adolescents’ psychological processes and mental health (Zhao & Li, 2022; Garandeau & Salmivalli, 2019). Nevertheless, further investigation is required to clarify how these norms interact with individual psychological processes to shape depressive symptoms. Additionally, existing findings remain inconsistent, as illustrated by the coexistence of perspectives such as the Healthy Context Paradox and the peer contagion hypothesis. The present study aims to advance the field through several contributions: First, it tests a moderated mediation model that incorporates cognitive biases as the individual pathway linking peer victimization to depressive symptoms, with clique victimization norms serving as a cross-level moderator. Second, it reconciles competing theoretical frameworks by elucidating how clique victimization norms moderate the association between victimization and cognitive biases. By integrating contextual and individual factors, this study deepens our understanding of how clique norms influence adolescent depressive symptoms.

### 1.4. Potential Gender Differences

Significant gender differences exist in how individuals experience peer victimization and depressive symptoms. Research shows that adolescent boys report more depressive symptoms and are more frequently bullied than girls (Gao et al., 2022; Garandeau et al., 2018). Victimized boys also exhibit more relational aggression in cliques with high victimization norms (Zhao & Li, 2022). Despite higher rates of victimization, some studies find no gender differences in depression (Akıncı & Güven, 2015). From mid-adolescence onward, girls are nearly twice as likely to develop depression, a disparity that persists into adulthood (Piccinelli & Wilkinson, 2000; Girgus & Yang, 2015). This increased vulnerability is likely due to greater cognitive susceptibility among girls (Bar-Tal & Jarymowicz, 2010).

Socio-cultural factors shape distinct gender roles, influencing emotional expression and stress management. Girls are encouraged to express emotions and seek support, while boys are expected to suppress emotions and solve problems independently (Rosenfield & Mouzon, 2013). Consequently, girls develop a heightened sensitivity to social exclusion or status loss, which is often intensified by societal norms. This sensitivity increases their risk of depression in bullying contexts (Cyranowski et al., 2000). However, the role of cognitive biases and clique victimization norms in these gender-specific patterns remains understudied. Therefore, this study aims to explore how clique dynamics and cognitive processes contribute to gender differences in depressive symptoms.

### 1.5. Current Study and Depression in Chinese Culture

**Figure 1:**
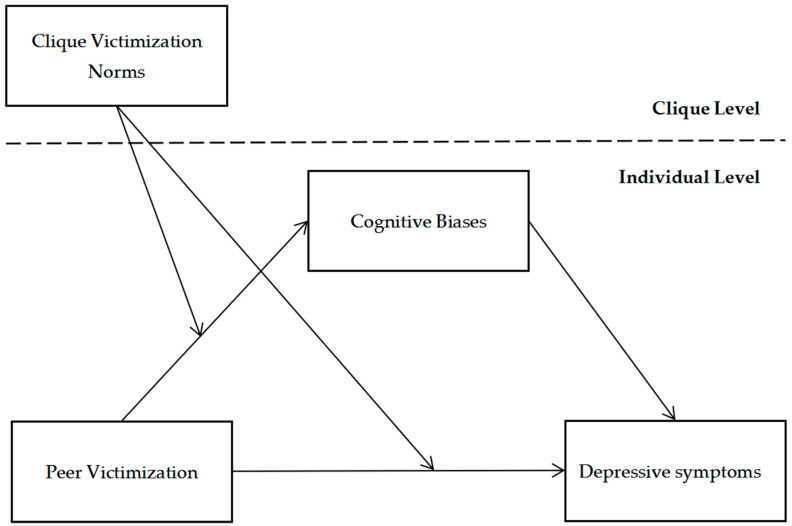
The proposed multilevel moderated mediation model.

To address the growing risk of depression in adolescents, which is often insufficiently understood in Chinese society, this study analyzed baseline data (the first wave of a two-year longitudinal study) to examine the association between peer victimization and adolescent depressive symptoms, particularly within cliques. This study also sought to raise awareness of emotional changes among bullying victims. In China, depressive symptoms are often misinterpreted as incompetence or inappropriate expressions of negative emotions. Using a sample of junior middle school students, this study incorporated cognitive biases as an individual-level variable within a mediation framework. This approach allowed for an examination of whether the observed associations align with Beck’s Model (Aim 1). Additionally, “clique victimization norms” were introduced as a moderator to examine the association between victimization norms and the mediating role of cognitive biases (Aim 2). Gender differences in these mediation-related associative patterns were also explored (Aim 3). The research framework is outlined in Figure 1.

Based on this framework, the following hypotheses were proposed: (1) Cognitive biases will be positively associated with depressive symptoms in peer-victimization contexts (Hypothesis 1); (2) clique victimization norms will moderate the association between peer victimization and cognitive biases (Hypothesis 2); (3) the proposed associative patterns will differ across genders (Hypothesis 3).

## 2. Methods

### 2.1. Participants and Procedures

Data collection took place in May 2015 across three junior high schools in Xi’an, Shaanxi Province, involving 2647 students. Two weeks prior to testing, we contacted the schools and, after obtaining school approval, used cluster sampling to select 16 seventh-grade classes. Informed consent forms were distributed, and students who signed them participated in the study. The paper-and-pencil questionnaire was administered under clear instructions, with students completing it independently within 30 min, and strict confidentiality of responses was ensured. After excluding 78 participants due to incomplete personal information, the final valid number of subjects was 2569 (53.4% boys, *M_age_* = 13.26 years, *SD* = 0.67). Using the Social Cognitive Map method, 2091 students (54.3% boys, *M_age_* = 13.26, *SD* = 0.66) were identified as members of cliques.

### 2.2. Measures

#### 2.2.1. Cognitive Biases

The Children’s Negative Cognitive Error Questionnaire (Leitenberg et al., 1986) was conducted to assess students’ cognitive biases. The questionnaire is used to assess three types of cognitive errors in three areas of life (athletic, social, and academic). “Catastrophizing” (in the athletic area) anticipates that if the outcome of an event will be either exceedingly negative or catastrophic (e.g., your cousin calls you to ask if you would like to go on along bike ride. You think, “I probably won’t be able to keep up and people will make fun of me.”). “Selective abstraction” (in the athletic area) occurs when attention is fixed narrowly on the negative elements of an experience, neglecting the rather neutral or positive aspects (e.g., you play basketball and score 5 baskets but missed two real easy shots. After the game you think, “I played poorly”). “Personalizing” (in the social area) is an exaggeration of assuming personal responsibility for negative events or the interpretation of these events as if they had a personal meaning (e.g., you call one of the kids in your class to talk about your math homework. He/she says, “I can’t talk to your now, my father needs to use the phone.” You think, “He/she didn’t want to talk to me.”). “Over generalizing” (in the academic area) is the belief that a negative outcome would occur in any case if it occurred once even the cases share slight similarities (e.g., last week you had a history test and forgot some of the things you had read. Today you are having a meth test, and the teacher is passing out the test. You think, “I’ll probably forget what I studied just like last week.”). Students rated each item on a 5-point scale from 1 (not at all like I would think) to 5 (almost exactly like I would think). The Cronbach’s alpha for the total score was 0.89, the alphas for the three content areas ranged from 0.75 to 0.82.

#### 2.2.2. Peer Victimization

Data of peer victimization was assessed by in-class self-reports. And self-reported victimization was measured by employing 8 items (3 of physical victimization, 5 of relational victimization) from the Social Experiences Questionnaire (Crick & Grotpeter, 1996) and 2 items (verbal victimization) from the Chinese version of the Olweus Bully/Victim Questionnaire (Zhang & Wu, 1999). The questionnaire was rated on a 5-point scale from 0 (never) to 4 (more than five times) and the score of which was computed as a mean score indicating a positive prediction of victimization for each cub-scale. The questionnaire was proved to hold good internal reliability (α = 0.91) and acceptable structural validity (*χ*^2^/*df* = 3.805, *p* < 0.001, CFI = 0.980, TLI = 0.966, RMSEA = 0.070, 90% CI = [0.056, 0.085], SRMR = 0.030). The same data was well adapted to further analysis.

#### 2.2.3. Depressive Symptoms

The Center for Epidemiological Studies Depression Scale (CES-D; Radloff, 1977) was employed to evaluate students’ depressive symptoms in this study. The scaled consists four dimensions (i.e., “depressed affect”, “positive affect”, “somatic and retarded activity”, “interpersonal”). A total of twenty items were measured on a 5-point scale from 1 (none) to 4 (all the time) including item 8 (I felt hopeful about the future), and item 12 (I was happy), item 16 (I enjoyed life) with reverse scoring. Mean score was computed, and a higher score represents a higher depressive symptom. “Depressed affect” (e.g., I was bothered by things that usually don’t bother me) and “Positive affect” (e.g., I felt hopeful about the future) are meant to reflect students’ emotions and mental states. “Somatic and retarded activity” is the evaluation of students’ abnormal reactions or daily activities including appetite, effort, sleep and get going (e.g., I did not feel like eating; my appetite was poor). The “Interpersonal” dimension, with two items, is mainly used to assess the quality of students’ social connections (e.g., people were unfriendly; I felt that people dislike me). Cronbach’ s alpha coefficients were good at 0.88. KMO and Bartlett’s test of sphericity analysis were performed; the results showed that *p* < 0.001 and KMO = 0.93, and thus the validity was good.

#### 2.2.4. Peer Cliques

Students’ social cliques in classrooms were identified by employing the Social Cognitive Map procedure (SCM; Cairns et al., 1988), which has been proved to have good reliability and validity in Chinese culture environments (Chen & Chen, 2020). Cliques are small social groups formed by people who hang around a lot. Students were instructed to nominate their own social groups (e.g., Do you have a group that you often hang around with in your class? Who are these people?) and to report, with a maximum of 11, any other cliques in classrooms. Each clique contains 3 to 12 members. And students who did not participate were also allowed to be nominated for ensuring the validity. Principal components analysis (PCA) showed all-boy cliques (N = 1050, *M_age_* = 13.30 years, *SD* = 0.69), all-girl cliques (N = 881, *M_age_* = 13.21 years, *SD* = 0.64) and mixed-gender cliques (N = 160, 48.13% boys, *M_age_* = 13.63 years, *SD* = 0.63). To examine whether there were distinctions among participants who took part in the follow-up study and those who were absent, an attrition analysis was processed, and no gender differences were found (*χ*^2^ (1) = 2.447, *p* = 0.118, age, *t* (689) = 0.180, *p* = 0.858). Little’s Missing Completely at Random (MCAR) test was significant (*χ*^2^ (138) = 226.178, *p* < 0.05), suggesting the data were not missing completely at random. Full information maximum likelihood was employed to handle missing data before proceeding with the analysis.

#### 2.2.5. Clique Victimization Norms

Clique victimization norms were defined as the average level of self-reported or peer-nominated victimization within the clique (Zhao & Li, 2022). Clique victimization norms were calculated using self-reported rather than peer-nominated victimization data. This approach was chosen because self-reports more directly reflect individuals’ subjective perceptions, which serve as key pathway variables for cognitive and emotional outcomes, while also avoiding the biases and data incompleteness often present in peer nominations (Song et al., 2025; Zhao & Li, 2022).

### 2.3. Data Analysis

The preliminary analyses, including descriptive analysis, correlation analysis and independent-sample t-test, were performed in SPSS 29.0. Other analyses were performed with multilevel modeling in Mplus 8.3. A three-step method was conducted: First, the unconditional model with no predictors was used to obtain the ICCs of cognitive biases and depressive symptoms (see Section 3). Second, the individual-level mediation model was built to test the relationships among the variables. Third, clique-level variables were added to see how clique victimization norms further moderate the model.

## 3. Results

### 3.1. Preliminary Analyses

Table 1 presents descriptive statistics and correlation analysis. For boys (see Table 1 (a)), both peer-victimization and cognitive biases have a significant positive correlation with depressive symptoms, suggesting that higher levels of victimization and biases are associated with more depressive symptoms. And a significant positive correlation between peer-victimization and cognitive biases was detected, indicating that as peer victimization increases, cognitive biases also tend to increase. For girls (see Table 1 (a)), cognitive biases were found to have significant correlations with both age and peer-victimization, indicating individuals who are older and suffer from peer-victimization report more cognitive biases than others. Depressive symptoms’ strong correlations with peer-victimization and cognitive biases suggest that individuals who suffer from peer-victimization and have cognitive biases exhibit more depressive symptoms. Table 1 (b) presents the independent-sample t-test for the gender differences across all variables. The results showed that, except for age, significant gender differences were found for all three other variables. At clique level, clique size was larger for cliques with a higher proportion of boys. Clique victimization norms had non-significant correlations with clique size or clique proportion of boys (see Table 1 (c)).

### 3.2. The Unconditional Model

According to Cohen’s (1977) guidelines, the ICCs of cognitive biases (0.024) and depressive symptoms (0.028) were small (ICC < 0.059). Although the between-clique variability in cognitive biases and depressive symptoms was small, a two-level analysis was still conducted. Given that previous studies have also employed multilevel models for data analysis despite ICC values below 0.059 (Pan et al., 2021; Selig et al., 2008; Zhao & Li, 2022), our core interest lies in examining how clique-level victimization norms shape the associations between individual victimization and cognitive biases or depressive symptoms, rather than explaining variance in cognitive biases or depressive symptoms themselves.

### 3.3. The Individual-Level Mediating Model

**Figure 2:**
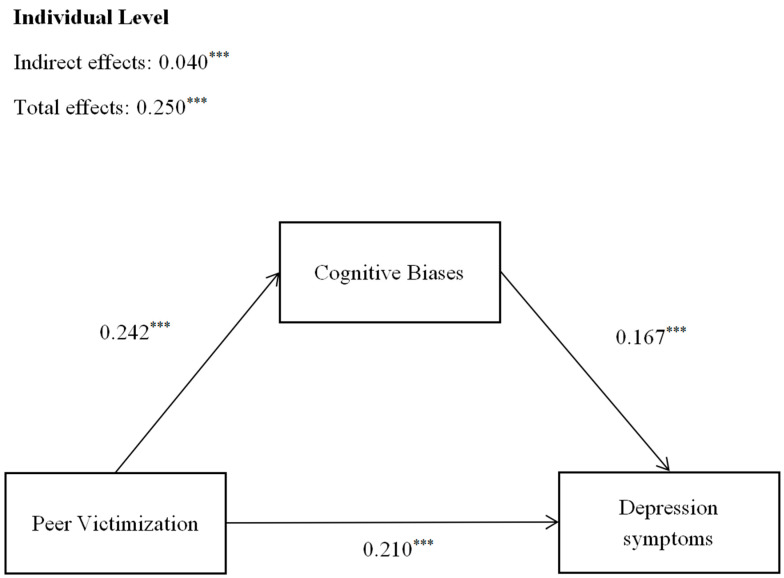
The mediating role of cognitive biases in the relationships between peer victimization and depressive symptoms. The models control age and gender, and the coefficients are for the models of depressive symptoms, *** *p* < 0.001.

The mediating effects of cognitive biases on the relationships between peer victimization and depressive symptoms without clique-level predictors are presented in Figure 2 and Table 2.

For the mediating effects of cognitive biases (see Figure 2 and Table 2), individuals who perceived more peer victimization reported more cognitive biases (*Bs* = 0.242, *SEs* = 0.027, *ps* < 0.001) and depressive symptoms (*Bs* = 0.210, *SEs* = 0.024, *ps* < 0.001). Cognitive biases were positively associated with individual depressive symptoms (*Bs* = 0.167, *SEs* = 0.017, *ps* < 0.001). The associative pathway involving cognitive biases was consistent with the proposed mediation framework and reached statistical significance (*Bs* = 0.040, *SEs* = 0.006, *ps* < 0.001), indicating that the associations between peer victimization and depressive symptoms were partly accounted by cognitive biases. The individual-level variables explained 10.34%, 7.56% and 18.37% of the variance in depressive symptoms.

### 3.4. The Clique-Level Model

Clique-level variables were further added to the model to examine their associations with the variables implicated in the mediation framework (i.e., cognitive biases) and the outcome variable (i.e., depressive symptoms), as well as the associative pattern between peer victimization and these variables (see Table 3 and Table 4).

**Figure 3:**
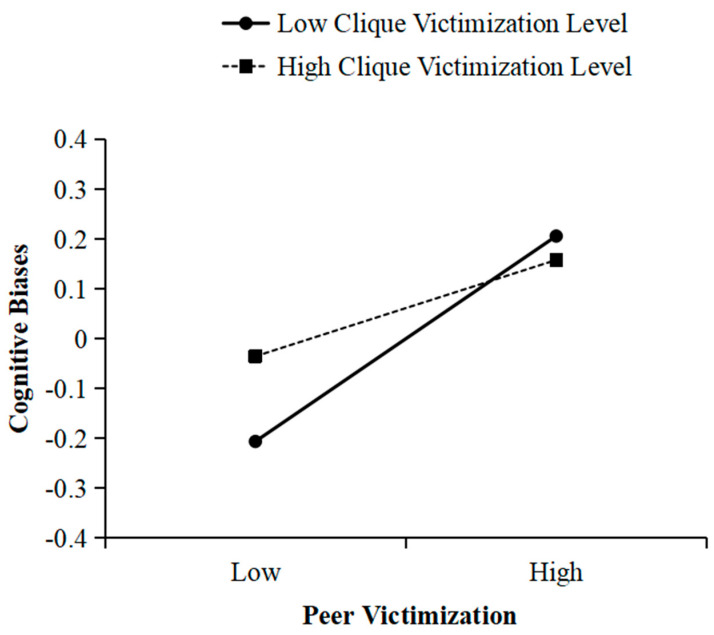
Clique-level victimization moderated the effect on cognitive biases from peer victimization.

For models incorporating cognitive biases as a variable implicated in the mediation framework (see Table 3), after controlling for clique boy proportion and clique size, clique victimization norms were positively associated with cognitive biases (*Bs* = 0.038~0.039, *SEs* = 0.014, *ps* < 0.01), but not depressive symptoms (*Bs* = 0.004~0.012, *SEs* = 0.008~0.012, *ps* > 0.05). There were marginally significant or significant two-way cross-level interactions between peer victimization and clique victimization norms for cognitive biases (*B* = −0.066, *SE* = 0.031, *p* < 0.05; *B* = −0.068, *SE* = 0.031, *p* < 0.05; *B* = −0.056, *SE* = 0.031, *p* = 0.069), rather than for depressive symptoms (*Bs* = −0.023~0.002, *SEs* = 0.018~0.021, *ps* > 0.05). The clique-level predictors explained 11.76%, 5.88%, and 10.52% of the variance in cognitive biases between cliques, and 31.25%, 23.53% and 18.75% of the variance in peer victimization, cognitive biases slops of the Model in Table 3 based on the cognitive bias slopes of the model shown in Table 3. To further probe these significant interactions, follow-up analysis of simple slopes was conducted for adolescents in cliques with either high (*M + 1SD*) or low (*M − 1SD*) victimization norms. As shown in Table 4 and Figure 3, peer victimization was more strongly associated with cognitive biases in low-victimization cliques (*Bs* = 0.340~0.351, *SEs* = 0.048~0.049, *p* < 0.001) than in high-victimization cliques (*Bs* = 0.192~0.208, *SEs* = 0.049, *p* < 0.001). Although the conditional effects were significant in both types of cliques, they were stronger in low-victimization cliques than in high-victimization cliques (Bmodel1-difference = 0.155, *SE* = 0.073, *p* = 0.035; Bmodel2-difference = 0.159, *SE* = 0.074, *p* = 0.031; Bmodel3-difference = 0.132, *SE* = 0.074, *p* = 0.073). Notably, while clique-level victimization norms significantly moderate the individual-level associations, the magnitude of these clique-level effects is modest (consistent with the low ICCs for cognitive biases and depressive symptoms).

### 3.5. Supplementary Analyses

Given that we found significant gender differences in the main study variables, whether the moderated mediating effects varied across genders was tested. The multilevel moderated mediating models were examined in all-girl cliques (n = 883), all-boy cliques (n = 1050), and mixed-gender cliques (n = 160), separately (see Table S1).

In models incorporating cognitive biases as a variable implicated in the mediation framework, similar associative patterns were observed in all-girl cliques (see Figure S1), indicating that girls’ victimization was positively associated with cognitive biases in all-girl cliques with low victimization norms (*Bs* = 0.448~0.468, *SEs* = 0.064~0.088, *ps* < 0.001), but not with high victimization norms (*Bs* = 0.115~0.148, *SEs* = 0.099~0.102, *ps* > 0.05), which, in turn, were positively associated with their depressive symptoms (*B* = 0.176, *SE* = 0.025, *p* < 0.001). In the all-boy clique model (see Figure S2), victimization was more strongly positively associated with cognitive biases in all-boy cliques with low victimization norms (*B* = 0.348, *SE* = 0.065, *p* < 0.001) than in all-boy cliques with high victimization norms (*B* = 0.198, *SE* = 0.056, *p* < 0.001). There were marginally significant differences between these two conditional effects (*B* = 0.50, *SE* = 0.090, *p* = 0.098). However, in the all-boy clique model, the multi-level moderated mediating effects on depressive symptoms were non-significant, and the same applied for models of mixed-gender cliques (see Table S1).

## 4. Discussion

The Healthy Context Paradox suggests that victims in cliques with lower victimization norms are more likely to experience increased depressive symptoms. This cross-sectional study supports this paradox and aligns with Beck’s Model. The findings indicate that the higher prevalence of depressive symptoms among victims may stem from biased cognitive processing. Beck’s Model emphasizes the critical role of cognitive processing in shaping responses to social interactions. The results suggest that in cliques with lower victimization norms, heightened cognitive biases contribute to more severe social exclusion and self-rejection, which amplifies victims’ feelings of humiliation and lack of peer acceptance. Consequently, victims may experience worsening depressive symptoms over time. These effects were consistent across cliques of varying sizes and gender ratios, although their manifestation differed based on clique gender composition.

### 4.1. The Effects of Cognitive Biases

The findings reveal a strong link between cognitive biases and peer victimization. Peer victimization also showed a significant positive association with depressive symptoms, an effect that was more pronounced in girls. According to Beck’s Model (Beck, 2008), victimization acts as a stressor that triggers cognitive schemas in victims, contributing to biased thinking and subsequent depressive symptoms. The severity of depressive symptoms depends on the type of cognitive schema activated and the lasting impact of bullying experiences.

The intensity of victimization correlates with psychological trauma, which in turn exacerbates depressive symptoms. Consistent with Beck’s Model, the data suggest that victimization alters victims’ emotional processing within cliques by shaping cognitive schemas that reflect perceived threat levels. These schemas foster cognitive biases, with victimization acting as the key trigger. Thus, the findings align with Beck’s Model, which provides a coherent theoretical framework for interpreting the associations among peer victimization, cognitive biases, and depressive symptoms.

### 4.2. The Interaction Effects of Individual and Clique Victimization

The results showed that peer victimization was more strongly associated with greater cognitive biases in cliques with low victimization norms. In these low-norm contexts, victimized adolescents also exhibited more depressive symptoms. These findings align with previous research (Zhao & Li, 2022) and support Hypothesis 2 and the Healthy Context Paradox (Garandeau & Salmivalli, 2019). The most robust evidence emerges for the moderation of the individual peer victimization and cognitive biases by clique-level norms, particularly in all-girl cliques. Specifically, victimization showed a stronger association with cognitive biases in low-victimization cliques, whereas this association was attenuated in high-victimization cliques. This nuanced pattern advances the Healthy Context Paradox. It clarifies that the paradox’s core effect, which is heightened vulnerability in low-victimization contexts, is driven by the amplification of cognitive biases rather than by a direct effect of the context on depressive symptoms. Correspondingly, victims in low-norm cliques reported more cognitive biases than their counterparts in high-norm cliques. Two mechanisms may explain this finding. First, in environments where victimization is uncommon, victims are more likely to attribute their mistreatment to internal, stable factors such as self-blame, especially when they perceive that peers are not similarly targeted (Schacter & Juvonen, 2015). This attributional style fosters persistent cognitive biases. Second, a prevalent social–cognitive belief casting bullies as “strong” and victims as “weak” may further inhibit self-defense. When bullying is rare within a group, each incident becomes more salient. This heightened salience can intensify victims’ fear, reduce peer acceptance, and lower self-esteem, thereby consolidating negative cognitive patterns.

It is noteworthy that clique victimization norms alone were not directly associated with depressive symptoms. This suggests that the influence of these norms operates primarily through an indirect pathway by shaping cognitive biases. In low-victimization cliques, the normative deviance of being victimized amplifies the internalization of adverse experiences. This occurs through specific cognitive processes, including personalization, which involves attributing victimization to internal traits, and social comparison, which reinforces a perception of being an outgroup member. These processes align with Beck’s model, as they activate vulnerable schemas and establish maladaptive information processing routines, thereby strengthening the link between victimization and depressive symptoms. Collectively, these insights extend the social–ecological perspective (Swearer & Espelage, 2004) by identifying a key cognitive pathway. They indicate that the likelihood of victimization leading to depression varies by context, with specific intermediary mechanisms, such as the cognitive biases outlined here, playing a critical role in certain settings.

Finally, the modest magnitude of the clique-level effects is consistent with the low ICCs. This reflects the multifactorial nature of adolescent mental health, which is shaped by a confluence of individual, family, and broader peer factors beyond a single clique. Therefore, while clique norms meaningfully moderate the specific pathway examined, they constitute one layer of influence among many. Consequently, interpretations should avoid overstating the strength of these group-level effects.

### 4.3. Gender Differences

The study examined differences across all-boy, all-girl, and mixed-gender cliques. The results support Hypothesis 3, indicating that girls’ depressive symptoms are more influenced by the Healthy Context Paradox than those of boys. In all-girl cliques with low victimization norms, victimization showed a stronger association with greater cognitive biases and, consequently, more depressive symptoms. This pattern aligns with the overall sample and supports Beck’s cognitive model. In contrast, while victimized boys in low-norm cliques also exhibited heightened cognitive biases, these biases did not lead to significantly greater depressive symptoms. This finding is consistent with prior research on gender differences in depression prevalence and expression (Bar-Tal & Jarymowicz, 2010; Girgus & Yang, 2015).

These gender-specific patterns can be understood by integrating psychological, physiological, and social factors. Psychologically, girls are more likely to engage in rumination (Feinstein et al., 2013) and make internal, stable, and global attributions for negative events (Hu et al., 2015). These cognitive styles may amplify and sustain cognitive biases following victimization (Cyranowski et al., 2000; Lewis et al., 2015), thereby strengthening the mediating role of such biases in the pathway to depressive symptoms. Physiologically, hormonal differences may contribute. For example, estrogen has been linked to heightened emotional reactivity in girls (Kuehner, 2017), whereas testosterone in boys may modulate stress responses differently. Socially, gender role expectations play a significant part (Eagly & Wood, 2012). Girls are often socialized to be more emotionally expressive and relationally sensitive, which may increase their vulnerability to interpersonal conflict and its associated cognitive biases (Berthet, 2021). Boys, conversely, may be encouraged to suppress emotional distress, potentially leading to a weaker observable link between cognitive biases and depressive outcomes.

Interestingly, no significant moderated mediation effects were found in mixed-gender cliques. This null finding may be attributed to the smaller sample size of such groups in our study, which limited statistical power. Additionally, the social dynamics in mixed-gender settings are more complex. Varied interactions and overlapping gender role expectations in these contexts may dilute or obscure effects that are more consistent in single-gender cliques.

### 4.4. Limitations and Future Directions

The study has several limitations related to data collection and measurement. First, regarding sampling, cluster sampling was adopted for its convenience and cost-efficiency, but this approach may lack the representativeness of random sampling. Clusters such as schools within the same city may not fully reflect the broader population of junior middle school students, potentially reducing the ecological validity of the findings. Additionally, the requirement for signed informed consent could introduce volunteer bias, meaning participants may differ from non-participants in factors such as socioeconomic status, which limits sample diversity. The focus on seventh-grade students also restricts generalizability, as psychological and behavioral traits vary across early adolescence.

A critical limitation is the cross-sectional design, which relies solely on baseline data. This design cannot establish temporal order among variables or support causal inferences. All key constructs were measured concurrently, so it remains unclear whether peer victimization precedes cognitive biases or vice versa. The observed associations reflect co-occurrence rather than causation. Another measurement limitation is that all key constructs were assessed via self-report at the same time point. This method raises the risk of common-method variance, which may inflate observed correlations. To address this, future research should integrate self-reports with alternative methods such as peer nominations or teacher reports. Furthermore, analyzing follow-up waves from the original longitudinal project would allow for longitudinal analyses, such as cross-lagged models, to clarify temporal dynamics and test potential causal pathways.

Second, the study has substantive limitations in exploring underlying mechanisms. While cognitive biases were implicated, this broad construct was not examined in terms of specific dimensions like catastrophizing or personalizing. Other relevant variables, such as perceived social support, family functioning, or coping strategies, were not included. These factors may interact with cognitive biases, suggesting that cognitive biases are unlikely to be the sole mechanism. Additionally, the specific role of peer acceptance in the relational pattern between victimization and depressive symptoms remains underexplored.

Finally, the mediation framework itself has inherent limitations. Focusing on a specific variable like cognitive biases narrows the scope for understanding depressive symptoms from a broader perspective. For instance, it remains unclear how the association between cognitive biases and depressive symptoms compares to that of other factors, such as family functioning or childhood trauma. Traditional mediation analyses may not fully address these comparative questions.

To address these limitations, future research should pursue several directions. Methodologically, studies should (1) adopt more representative sampling strategies; (2) combine self-reports with alternative measurement methods to mitigate common-method variance; and (3) conduct longitudinal analyses to examine temporal dynamics and causal directions. Substantively, future research should (1) explore specific dimensions of cognitive biases; (2) incorporate additional relevant variables like social support and family factors; (3) investigate the role of peer acceptance; and (4) examine the relevance of Beck’s Model to other maladaptive behaviors, such as aggression, to expand theoretical implications for adolescent social–emotional development.

### 4.5. Strengths and Implications

Despite its limitations, this study offers several strengths and important implications. First, it is among the first to examine the mediating role of cognitive biases in the link between peer victimization and depressive symptoms from a clique-level perspective. Second, it clarifies how cognitive biases shape this link through Beck’s Model, thereby integrating insights from social, developmental, and clinical psychology. This integration contributes to future interdisciplinary research. Third, given the scarcity of clique-level studies on victimization, the findings provide a valuable foundation and encourage further exploration.

Regarding practical implications, the findings suggest actionable directions for school-based interventions. Specifically, interventions could (1) monitor clique-level dynamics, with particular attention to low-victimization contexts where isolated victims may face heightened risk; (2) incorporate cognitive-focused strategies, such as evidence-based social–emotional learning or cognitive–behavioral programs adapted for early adolescents, to address the biases implicated in the victimization–depression association; and (3) prioritize support for victims in low-victimization cliques, where social disconnection may amplify cognitive vulnerabilities. These targeted strategies align with the study’s focus on cognitive and contextual factors and can help translate research into practice to support adolescent mental health.

## 5. Conclusions

Drawing on data from both individuals and their peer cliques, this study aimed to clarify the role of cognitive biases in the association between peer victimization and depressive symptoms. The results indicated that clique-level victimization norms operate in a manner consistent with the Healthy Context Paradox. Furthermore, the mediating role of cognitive biases aligns with and supports Beck’s Model. These findings suggest that bullying interventions should consider clique-level norms. Accordingly, educators and school mental health services should focus on maintaining children’s psychological health within these specific social contexts. Future research should investigate both internalizing and externalizing problems concurrently to provide a more comprehensive understanding of adolescent adjustment.

## Figures and Tables

**Table 1:** (**a**) Descriptive statistics and correlations of individual-level variables for boys and for girls; (**b**) independent-sample t-test for gender differences; (**c**) descriptive statistics and correlations of clique-level variables.

(a)										
	*M*	*SD*		1		2		3		4
boys										
1 Age	13.28	0.68		1						
2 Peer Victimization	0.48	0.75		−0.03		1				
3 Cognitive Biases	2.33	0.66		0.01		0.27 **		1		
4 Depressive Symptoms	1.81	0.45		0.05		0.37 **		0.28 **		1
girls										
1 Age	13.24	0.65		1						
2 Peer Victimization	0.34	0.56		−0.45		1				
3 Cognitive Biases	2.42	0.63		0.11 **		0.20 **		1		
4 Depressive Symptoms	1.86	0.39		0.05		0.38 **		0.34 **		1
(b)										
	*t*	*df*		*p*		99%CI				Cohen’s d
						LB		UB		
Age	2.159	2526.982		0.300		−0.011		0.126		0.672
peer-victimization	−2.705	2256.834		0.007		−0.096		−0.002		0.434
cognitive biases	5.115	2179.764		<0.001		0.071		0.215		0.672
depressive symptoms	−3.032	2223.010		0.002		−0.154		−0.012		0.652
(c)										
	*M*		*SD*		1		2		3	
1 Clique Proportion of Boys	0.52		0.48		1					
2 Clique Size	4.64		1.64		0.12 **		1			
3 Clique Victimization Norms	1.60		1.19		0.02		0.02		1	

Notes: ** *p* < 0.01.

**Table 2:** The individual-level mediating model (N = 2091).

	Cognitive Biases		Depressive Symptoms	
	*B*	*95% CI*	*B*	*95% CI*
Peer Victimization	0.242 ***	[0.199, 0.288]	0.210 ***	[0.171, 0.249]
Cognitive Biases	-	-	0.167 ***	[0.139, 0.195]
Mediation effect	-	-	0.040 ***	[0.031, 0.052]

*** *p* < 0.001.

**Table 3:** Multilevel regressions of peer victimization on depressive symptoms via cognitive biases (N = 2091).

	The Clique-Level Model	
	Cognitive Biases	Depressive Symptoms
	*B (SE)*	*B (SE)*
Individual Level		
Intercept	2.380 (0.017) ***	1.835 (0.011) ***
Gender	−0.126 (0.128)	−0.145 (0.073) *
Age	0.036 (0.043)	−0.007 (0.025)
Peer Victimization	0.273 (0.033) ***	0.205 (0.024) ***
Cognitive Biases	-	0.148 (0.018) ***
Clique Level		
Clique Boy Proportion	−0.075 (0.035) *	−0.040 (0.023) ^†^
Clique Size	−0.003 (0.009)	−0.003 (0.006)
Clique Victimization Norms	0.038 (0.014) **	0.006 (0.009)
Cross-Level Interaction		
Victimization × Clique Boy Proportion	−0.005 (0.067)	0.002 (0.021)
Victimization × Clique Size	0.005 (0.016)	−0.011 (0.012)
Victimization × Clique Victimization Norms	−0.056 (0.031) ^†^	0.002 (0.021)
Random Effect		
Residual (σ^2^)	0.371 (0.015) ***	0.133 (0.005) ***
Intercept (τ00)	0.017 (0.008) *	0.017 (0.004) ***
Slope (τ01)	0.013 (0.015)	0.033 (0.008) ***

Note. Code for gender: boys = 1, girls = 0. Low = one standard deviation below the mean; high = one standard deviation above the mean. ^†^ *p* < 0.10, * *p* < 0.05, ** *p* < 0.01, *** *p* < 0.001.

**Table 4:** Simple slope analysis results.

	Model
	*B (SE)*
Low Victimization Norms	0.340 (0.049) ***
High Victimization Norms	0.208 (0.049) ***
Difference between the two conditional effects	0.132 (0.074) ^†^

Note. Low = one standard deviation below the mean; high = one standard deviation above the mean. ^†^ *p* < 0.10, *** *p* < 0.001.

## Data Availability

The data presented in this study are available on request from the corresponding author.

## Supplementary Materials

The following supporting information can be downloaded at https://www.mdpi.com/article/10.3390/bs16010068/s1: Table S1: Multilevel Regressions of Peer Victimization on Depressive Symptoms via Cognitive Biases in Different Cliques; Figure S1: Clique-level victimization moderated the effect on girls’ cognitive biases from peer victimization in all-girl cliques; Figure S2: Clique-level victimization moderated the effect on boys’ cognitive biases from peer victimization in all-boy cliques.
