# Supplementary material for: Do Peer Cliques and Gender Differences Shape Adolescent Depression Under Bullying? Exploring the Mediating Power of Cognitive Biases
Source: Behav Sci (Basel). 2026 Jan 4;16(1):68. doi: 10.3390/bs16010068 (PMC12838078; doi:10.3390/bs16010068)
Supplement: Supplementary file 1 [file behavsci-16-00068-s001.zip › Figure S2.pdf]

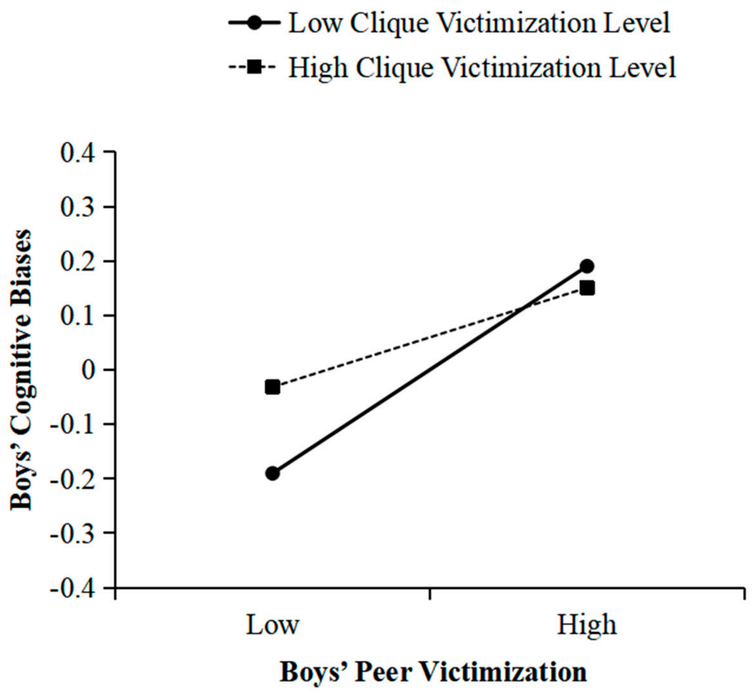

Figure S2. Clique-level victimization moderated the effect on boys' cognitive biases from peer victimization in all-boys cliques.
