# Supplementary material for: Do Peer Cliques and Gender Differences Shape Adolescent Depression Under Bullying? Exploring the Mediating Power of Cognitive Biases
Source: Behav Sci (Basel). 2026 Jan 4;16(1):68. doi: 10.3390/bs16010068 (PMC12838078; doi:10.3390/bs16010068)
Supplement: Supplementary file 1 [file behavsci-16-00068-s001.zip › Table S1.pdf]

Table S1

Multilevel Regressions of Peer Victimization on Depressive Symptoms via Cognitive Biases in Different Cliques

	**All-Boys Cliques (*n* = 1050)**		**All-Girls Cliques (*n* = 881)**		**Mixed-Gender Cliques (*n* = 160)**	
	Cognitive Biases	Depressive Symptoms	Cognitive Biases	Depressive Symptoms	Cognitive Biases	Depressive Symptoms
	B (SE)	*B (SE)*	*B (SE)*	*B (SE)*	*B (SE)*	*B (SE)*
**Individual Level**						
Intercept	2.345 (0.025)***	1.809 (0.017)***	2.434 (0.024)***	1.857 (0.015)***	2.345 (0.072)***	1.909 (0.053)***
Age	0.086 (0.059)	-0.054 (0.037)	-0.017 (0.066)	-0.098 (0.042)	0.044 (0.158)	0.065 (0.099)
Peer Victimization	0.256 (0.039)***	0.181 (0.032)***	0.303 (0.046)***	0.225 (0.040)***	0.269 (0.079)**	0.251 (0.071)***
Cognitive Biases	-	0.146 (0.028)***	-	0.176 (0.025)***	-	0.081 (0.063)
**Clique Level**						
Clique Size	0.007 (0.012)	0.000 (0.008)	-0.027(0.016)	-0.014 (0.010)	-0.003 (0.045)	0.012 (0.034)
Clique Victimization Norms	0.035 (0.021)†	0.011 (0.014)	0.040 (0.019)*	0.006 (0.012)	0.075 (0.066)	-0.012 (0.048)
**Cross-Level Interaction**						
Victimization × Clique Size	0.019 (0.018)	-0.014 (0.015)	-0.040 (0.040)	-0.009 (0.024)	0.022 (0.057)	-0.001 (0.045)

Victimization × Clique Victimization Norms	-0.051 (0.039)	0.012 (0.030)	-0.135 (0.058)*	0.018 (0.033)	0.120 (0.102)	-0.063 (0.083)
**Random Effect**						
Residual (σ^2)	0.368 (0.022)***	0.150 (0.009)***	0.361 (0.023)***	0.107 (0.007)***	0.411 (0.058)***	0.169 (0.023)***
Intercept ($\tau00$)	0.020 (0.012)	0.016 (0.006)***	0.008 (0.012)	0.011 (0.005)*	0.052 (0.050)	0.057 (0.028)*
Slope ($\tau01$)	0.008 (0.019)	0.035 (0.011)***	0.028 (0.038)	0.021 (0.019)*	0.002 (0.017)	0.044 (0.023)†
**Simple Slope Analyses**						
Low Victimization Norms[a]	-	-	0.468 (0.064)***	-	-	-
High Victimization Norms[a]	-	-	0.139 (0.102)	-	-	-

Note. [a]low = one standard deviation below the mean; high = one standard deviation above the mean

†$p < 0.10$, *$p < 0.05$, **$p < 0.01$, ***$p < 0.001$
